# Dietary diversity and associated factors among infants and young children in three West African countries

**DOI:** 10.3389/fpubh.2024.1386664

**Published:** 2024-07-24

**Authors:** Amynah Janmohamed, Melissa M. Baker, David Doledec, Fatou Ndiaye, Ahmenan Claude Liliane Konan, Amoakon Leonce, Koffi Landry Kouadio, Maguette Beye, Mohamed L. Yattara, Romance Dissieka

**Affiliations:** ^1^Helen Keller International, Vitamin A Supplementation Africa Regional Office, Nairobi, Kenya; ^2^Helen Keller International, Abidjan, Côte d'Ivoire; ^3^Helen Keller International, Dakar, Senegal; ^4^Helen Keller International, Niamey, Niger

**Keywords:** Africa, child, dietary diversity, food groups, nutrition

## Abstract

Providing children healthy diversified diets is important for their optimal growth and development. The high prevalence of under-nourishment during the critical early life period is of serious concern in West Africa. We assessed the level of dietary diversity and associated factors for children aged 6–23 months in Côte d’Ivoire, Niger and Senegal. Prior 24 h dietary intake was assessed for 3,528 children (Côte d’Ivoire: *N* = 118; Niger: *N* = 763; Senegal: *N* = 2,647) using the Diet Quality Questionnaire survey tool administered to primary caregivers. Cluster random sampling was conducted for urban and rural areas in Niger and Senegal and simple random sampling was used in Côte d’Ivoire, where only rural households were selected. Survey data were analyzed to determine children’s intake of items from eight food groups: breast milk; grains, roots, tubers and plantains; pulses, nuts and seeds; dairy products; flesh foods; eggs; vitamin A-rich fruits and vegetables; and other fruits and vegetables. Minimum Dietary Diversity (MDD) was assessed based on the consumption of ≥5 of the 8 food groups. In all countries, the majority of children were ≥ 12 months of age and from rural households. Children from poor/very poor households ranged from 32.4 to 41.9%. MDD prevalence was 54.2% in Côte d’Ivoire, 33.3% in Niger and 30.8% in Senegal. In all three countries, children 12–23 months had significantly higher consumption of six of the food groups, compared to those 6–11 months, and children ≥12 months had a higher likelihood of MDD, compared to infants, in Niger (aOR = 4.25; 95% CI: 2.46, 7.36) and Senegal (aOR = 2.69; 95% CI: 2.15, 3.35). MDD prevalence was higher among children in urban, compared to rural, areas in Niger (*p* = 0.020) and Senegal (*p* < 0.001) and significantly higher in the wealthiest, compared to poorest, households. This study suggests most young children in Côte d’Ivoire, Niger and Senegal are not receiving an adequately diversified diet, with a reliance on starchy staples and lower intake of high-quality protein sources. Our results highlight socio-economic barriers to attaining dietary diversity in these settings and stress the urgent and continuing need for investments in strategies that support optimal complementary feeding practices.

## 1 Introduction

Providing children a diversified and well-balanced diet containing essential nutrients is critical for their survival and optimal growth and development (1, 2). Good infant and young child feeding (IYCF) practices, including an adequately diversified diet, from birth to 2 years of age are particularly important for healthy outcomes into childhood and later life (3). The associations between nutritionally-varied diets and reduced risks of stunting, wasting and underweight are evident among children aged 6–23 months in sub-Saharan Africa (SSA) (4, 5). Poor micronutrient intake during this critical period remains of particular concern in many countries, with recent global estimates indicating more than half of preschool-aged children have at least one micronutrient deficiency (5, 6). Though many countries have age-specific IYCF guidelines as part of national policies and strategies to reduce child malnutrition, adhering to recommended practices poses major challenges for populations in areas where a lack of food availability or affordability limits access to a variety of nutrition-promoting foods. Globally, ~30% of children aged 6–23 months have a minimum level of dietary diversity (7), with particular challenges surrounding the timely introduction of nutritious complementary foods at 6 months of age (8).

Despite decades-long country investments to achieve global nutrition targets, the under-nourishment of many young children during critical periods remains a serious public health concern in SSA, with estimates indicating only one-quarter of young children achieve an adequately diversified diet (4, 9–11). A study examining the diets of children aged 6–23 months in 33 SSA countries revealed 63% had insufficient intake of flesh foods and 52 and 81% had inadequate consumption of vitamin A-rich fruits and vegetables and other fruits and vegetables, respectively (12). For Western Africa, available evidence indicates 67% of children under 5 years have one or more micronutrient deficiencies and < 10% attain a minimum acceptable diet as defined by the World Health Organization (WHO) (5, 11). Additional estimates suggest approximately 40% of children aged 6–23 months consume flesh foods as part of their daily diet in the West African region (5). Moreover, lower dietary diversity among infants 6–11 months, compared to those older than 1 year, has been widely observed and attributed to a greater reliance on breastmilk during infancy (8).

Given the critical role of healthy diets in integrated strategies to reduce child undernutrition, global dietary recommendations for infants and young children include exclusive breastfeeding for the first 6 months, continued breastfeeding until 24 months, and starting semi-solid/solid complementary foods at 6 months with progressive increases in variety, quantity and feeding frequency (3). Minimum dietary diversity (MDD) is one of 17 indicators developed by the WHO to assess the quality of IYCF practices at the household level and is based on a child’s consumption of foods from a minimum of five out of eight specific food groups the previous day: breast milk; grains, roots, tubers and plantains; pulses, nuts and seeds; dairy products; flesh foods; eggs; vitamin A-rich fruits and vegetables; and other fruits and vegetables (3). MDD is considered a proxy for adequate micronutrient intake based on the assumption that, in addition to a starchy staple food (grain, root, tuber), children attaining MDD likely consumed at least one animal-source food and one fruit or vegetable during the previous day (13).

Given the high burden of micronutrient malnutrition in Western Africa, there is a need for timely knowledge of young children’s diets to assess their nutritional adequacy and target appropriate interventions to the most vulnerable groups. The objective of this study was to assess the level of dietary diversity and associated factors for children aged 6–23 months in Côte d’Ivoire, Niger and Senegal.

## 2 Materials and methods

### 2.1 Sampling and data collection

Children’s prior 24 h dietary intake was assessed using Diet Quality Questionnaires (DQQ) administered to primary caregivers at the household. The DQQ is a standardized low-burden list-based tool for collecting data on consumption of healthy and unhealthy foods and has been adapted for use in numerous countries (14–16). The IYCF DQQ aligns with the WHO IYCF guidance (3) and is being incorporated into Demographic and Health Surveys (DHS) (14). Data collection took place during the lean season in Senegal (August 2022), during the transition period between full-season harvest and the start of sowing season in Côte d’Ivoire (November 2022), and during the harvest season in Niger (January 2023). In Niger and Senegal, survey regions were stratified into rural and urban zones and enumeration areas (EA) were selected using probability proportional to size sampling in accordance with standard sample size calculations. In Niger, 60 EAs were selected in both rural and urban areas in seven of the eight country’s regions. In Senegal, 366 EAs were selected from rural and urban areas in three regions (Diourbel, Louga, Tambacounda). Households were randomly selected from lists for each EA and the surveyed samples were representative of rural and urban settings in the respective regions. In Côte d’Ivoire, 20 rural EAs were included in one northern region (Poro) that were part of an ‘Enhanced Homestead Food Production’ project implemented by Helen Keller Intl and Latter-day Saint Charities focused on improving the nutrition, health and hygiene of women of childbearing age and mothers of children under 2 years. Survey eligibility was based on a child aged 6–23 months residing in the household and a primary caregiver being present at the time of the household visit. The child’s age was obtained from the caregiver and verified with the child’s health card, if available. Survey enumerators were recruited and trained on the DQQ methodology by Helen Keller Intl and collaborating partners and administered surveys in local languages. Data were recorded using secured mobile tablets. Research ethics approval was obtained from Comité National d’Ethique des Sciences de la Vie et de la Santé in Côte d’Ivoire, Comité National d’Ethique pour la Recherche en Santé in Niger, and Comité National d’Ethique pour la Recherche en Santé in Senegal. Written informed consent was obtained in Niger and Senegal and verbal informed consent was obtained from caregiver survey participants in Côte d’Ivoire.

### 2.2 Data analysis

Data were analyzed to determine children’s prior day consumption of foods from eight food groups defined by the WHO for assessing infant and young child feeding practices: breast milk; grains, roots, tubers and plantains; pulses, nuts and seeds; dairy products; flesh foods; eggs; vitamin A-rich fruits and vegetables; and other fruits and vegetables (3). In accordance with the DQQ methodology (14), each food group was coded with a binary score (0, 1) based on whether the child consumed at least one item from the group. The number of food groups consumed was summed to produce a score of 0–8 for each child. The primary study outcome was MDD, defined by the WHO as the proportion of children aged 6–23 months who consumed foods from ≥5 of the 8 food groups during the previous day or night (3). Secondarily, prior 24 h consumption of unhealthy beverages (e.g., sugary drinks) and processed snacks (e.g., chips) was recorded. Child, caregiver and household socio-demographic characteristics were assessed. Household wealth quintiles were created using principal components analysis based on a composite measure of asset ownership, housing characteristics and types of water access and sanitation facilities.

Bivariate analyses were conducted for data from all three countries and, for Niger and Senegal, multivariable regression models were used to explore associations between MDD and child age and sex, maternal education, rural or urban residence and household wealth. Model covariates were selected based on a significant (*p* < 0.05) bivariate result and/or previously acknowledged relationship with children’s diets. Weighted estimates accounted for the cluster sampling in Niger and Senegal. Due to the small sample size, a regression analysis was not performed for Côte d’Ivoire. All analyses were conducted using SPSS Complex Samples 26.0 (IBM Corp: Armonk, NY) with statistical significance assessed at *p* < 0.05. Results are presented as descriptive statistics and unadjusted and adjusted odds ratios with 95% confidence intervals in separate analyses for each country. The study was not designed to detect inter-country MDD differences.

## 3 Results

A total of 3,528 children aged 6–23 months were included in the study (Côte d’Ivoire: *N* = 118; Niger: *N* = 763; Senegal: *N* = 2,647; Table 1). The majority of children were ≥ 12 months of age in all countries, with approximately two-thirds aged 12–23 months in Niger (68.4%) and Senegal (65.4%). In Niger and Senegal, 52.4 and 80.0% of children resided in rural households, respectively. The proportions of children from very poor or poor households were 41.9, 32.4 and 37.5% in Côte d’Ivoire, Niger and Senegal, respectively (Table 1).

**Table 1:** Child characteristics.

	Côte d’Ivoire^†^ *N* = 118	Niger *N* = 763	Senegal *N* = 2,647
	n (%)	n (%)	n (%)
Area Rural Urban	118 (100.0) 0 (0.0)	400 (52.4) 363 (47.6)	2,118 (80.0) 529 (20.0)
Child age 6–11 months 12–23 months	54 (45.8) 64 (54.2)	241 (31.6) 522 (68.4)	915 (34.6) 1,732 (65.4)
Child sex Female Male	--- ---	380 (49.8) 383 (50.2)	1,274 (48.1) 1,373 (51.9)
Household wealth Very poor Poor Medium Rich Very rich	27 (23.1) 22 (18.8) 19 (16.2) 21 (17.9) 28 (23.9)	93 (12.2) 154 (20.2) 53 (6.9) 103 (13.5) 360 (47.2)	490 (18.5) 504 (19.0) 546 (20.6) 570 (21.5) 537 (20.3)

† In Côte d’Ivoire child sex data were not collected and only rural areas were targeted.

The majority of children in all countries consumed breast milk and grains, roots, tubers or plantains the previous day and about half consumed at least one vitamin A-rich fruit or vegetable (Table 2). Dairy product intake was 42.4% in Côte d’Ivoire, 42.5% in Niger and 52.4% in Senegal. Flesh foods were consumed by 52.5, 24.2 and 45.3% of children in Côte d’Ivoire, Niger and Senegal, respectively. Egg consumption was <20% in all three countries. Over half of the children in Côte d’Ivoire (54.2%) and about one-third of the children in Niger (33.3%) and Senegal (30.8%) were provided a minimally diverse diet (≥ 5 food groups) the previous day. Low consumption of sugary soft drinks was reported in all three countries (Côte d’Ivoire: 6.8%; Niger: 2.2%; Senegal: 2.9%). Children’s intake of packaged snacks was similar to that of sugary drinks in Côte d’Ivoire (6.8%) and Niger (2.4%), while substantially higher in Senegal (34.1%; Table 2).

**Table 2:** Food groups consumed and minimum dietary diversity for children aged 6–23 months.

	Côte d’Ivoire *N* = 118	Niger *N* = 763	Senegal *N* = 2,647
	n (%)	n (%)	n (%)
Breast milk	94 (79.7)	489 (64.1)	1,854 (70.0)
Grains, roots and tubers	88 (74.6)	592 (77.6)	2,267 (85.6)
Legumes and nuts	63 (53.4)	345 (45.2)	728 (27.5)
Dairy products	50 (42.4)	324 (42.5)	1,386 (52.4)
Flesh foods	62 (52.5)	185 (24.2)	1,200 (45.3)
Eggs	23 (19.5)	95 (12.5)	199 (7.5)
Vitamin A-rich fruits and vegetables	69 (58.5)	410 (53.7)	1,410 (53.3)
Other fruits and vegetables	75 (63.6)	341 (44.7)	659 (24.9)
Minimum Dietary Diversity^†^	64 (54.2)	254 (33.3)	816 (30.8)
Mean dietary diversity score^‡^	4.44 (4.08, 4.81)	3.64 (3.51, 3.78)	3.67 (3.61, 3.73)
Unhealthy foods Sugary drinks (e.g., soda) Processed snack foods (e.g., chips)	8 (6.8) 8 (6.8)	17 (2.2) 18 (2.4)	76 (2.9) 902 (34.1)

† Based on the child’s prior 24-h consumption of ≥ 5 of 8 food groups: breast milk; grains, roots and tubers; legumes and nuts; dairy products; flesh foods; eggs; vitamin A-rich fruits and vegetables; and other fruits and vegetables.

‡ Average number of food groups consumed (range 0–8).

The percentage of children provided breast milk the previous day was substantially higher among infants aged 6–11 months, compared to children 12–23 months, in all three countries (Côte d’Ivoire: 96.3% vs. 65.6%; *p* < 0.001; Niger: 83.8% vs. 55.0%; *p* < 0.001; Senegal: 96.0% vs. 56.4%; *p* < 0.001; Tables 3–5). In all three countries, the consumption of grains/roots/tubers/plantains, pulses/nuts/seeds, flesh foods, dairy products, vitamin A-rich fruits/vegetables, and other fruits/vegetables was significantly higher among children aged 12–23 vs. 6–11 months. MDD prevalence was also higher among children aged 12–23 months, compared to those aged 6–11 months, in all three countries (Côte d’Ivoire: 68.8% vs. 37.0%; *p* = 0.001; Niger: 40.8% vs. 17.0%; *p* < 0.001; Senegal: 37.2% vs. 18.7%; *p* < 0.001; Tables 3–5).

**Table 3:** Food group consumption and minimum dietary diversity by child age group and household wealth in Côte d’Ivoire^†^.

	Total	6–11 mo	12–23 mo	Poor	Medium	Rich
	n (%)	n (%)	n (%)	n (%)	n (%)	n (%)
Breast milk	94 (79.7)	52 (96.3)	42 (65.6)	36 (73.5)	18 (94.7)	40 (81.6)
Grains, roots, tubers	88 (74.6)	28 (51.9)	60 (93.8)	41 (83.7)	13 (68.4)	33 (67.3)
Pulses, nuts, seeds	63 (53.4)	16 (29.6)	47 (73.4)	32 (65.3)	7 (36.8)	23 (46.9)
Dairy products	50 (42.4)	21 (38.9)	29 (45.3)	19 (38.8)	6 (31.6)	24 (49.0)
Flesh foods	62 (52.5)	19 (35.2)	43 (67.2)	26 (53.1)	9 (47.4)	26 (53.1)
Eggs	23 (19.5)	11 (20.4)	12 (18.8)	9 (18.4)	3 (15.8)	11 (22.4)
Vitamin A-rich fruits / vegetables	69 (58.5)	20 (37.0)	49 (76.6)	34 (69.4)	7 (36.8)	27 (55.1)
Other fruits / vegetables	75 (63.6)	25 (46.3)	50 (78.1)	34 (69.4)	11 (57.9)	29 (59.2)
Minimum Dietary Diversity ^‡^	64 (54.2)	20 (37.0)	44 (68.8)	32 (65.3)	6 (31.6)	25 (51.0)

† Child age: breast milk 6–11 vs.12–23 *p* < 0.001; grains/roots/tubers 6–11 vs.12–23 *p* < 0.001; legumes/nuts 6–11 vs. 12–23 *p* < 0.001; flesh foods 6–11 vs.12–23 *p* = 0.001; vitamin A-rich fruits/vegetables 6–11 vs.12–23 *p* < 0.001; other fruits/vegetables 6–11 vs.12–23 *p* < 0.001; MDD 6–11 vs. 12–23 *p* = 0.001. Wealth status: vitamin A-rich fruits/vegetables poor vs. medium *p* = 0.039; MDD poor vs. medium *p* = 0.033.

‡ Based on the child’s prior 24-h consumption of ≥ 5 of 8 food groups: breast milk; grains, roots and tubers; legumes and nuts; dairy products; flesh foods; eggs; vitamin A-rich fruits and vegetables; and other fruits and vegetables.

**Table 4:** Food group consumption and minimum dietary diversity by child age group, sex, area and household wealth in Niger^†^.

	Total	6–11 mo	6–8 mo	9–11 mo	12–23 mo	Female	Male	Rural	Urban	Poor	Medium	Rich
	n (%)	n (%)	n (%)	n (%)	n (%)	n (%)	n (%)	n (%)	n (%)	n (%)	n (%)	n (%)
Breast milk	489 (64.1)	202 (83.8)	86 (85.1)	116 (82.9)	287 (55.0)	248 (65.3)	241 (62.9)	257 (64.3)	232 (63.9)	159 (64.4)	32 (60.4)	298 (64.4)
Grains, roots, tubers	592 (77.6)	150 (62.2)	52 (51.5)	98 (70.0)	442 (84.7)	287 (75.5)	305 (79.6)	301 (75.3)	291 (80.2)	185 (74.9)	44 (83.0)	363 (78.4)
Pulses, nuts, seeds	345 (45.2)	62 (25.7)	19 (18.8)	43 (30.7)	283 (54.2)	178 (46.8)	167 (43.6)	173 (43.3)	172 (47.4)	95 (38.5)	24 (45.3)	226 (48.8)
Dairy products	324 (42.5)	72 (29.9)	28 (27.7)	44 (31.4)	252 (48.3)	168 (44.2)	156 (40.7)	210 (52.5)	114 (31.4)	119 (48.2)	22 (41.5)	183 (39.5)
Flesh foods	185 (24.2)	29 (12.0)	8 (7.9)	21 (15.0)	156 (29.9)	88 (23.2)	97 (25.3)	46 (11.5)	139 (38.3)	28 (11.3)	7 (13.2)	150 (32.4)
Eggs	95 (12.5)	31 (12.9)	14 (13.9)	17 (12.1)	64 (12.3)	40 (10.5)	55 (14.4)	23 (5.8)	72 (19.8)	14 (5.7)	3 (5.7)	78 (16.8)
Vitamin A-rich fruits / veg	410 (53.7)	85 (35.3)	32 (31.7)	53 (37.9)	325 (62.3)	196 (51.6)	214 (55.9)	211 (52.8)	199 (54.8)	130 (52.6)	28 (52.8)	252 (54.4)
Other fruits / veg	341 (44.7)	78 (32.4)	27 (26.7)	51 (36.4)	263 (50.4)	173 (45.5)	168 (43.9)	150 (37.5)	191 (52.6)	90 (36.4)	20 (37.7)	231 (49.9)
Minimum Dietary Diversity ^‡^	254 (33.3)	41 (17.0)	12 (11.9)	29 (20.7)	213 (40.8)	130 (34.2)	124 (32.4)	118 (29.5)	136 (37.5)	65 (26.3)	14 (26.4)	175 (37.8)

† Child age: breast milk 6–8 vs. 12–23 *p* < 0.001 and 9–11 vs. 12–23 *p* < 0.001; grains/roots/tubers 6–8 vs. 9–11 *p* = 0.001, 6–8 vs. 12–23 *p* < 0.001 and 9–11 vs. 12–23 *p* < 0.001; legumes/nuts 6–8 vs. 12–23 *p* < 0.001 and 9–11 vs. 12–23 *p* < 0.001; dairy 6–8 vs. 12–23 *p* < 0.001 and 9–11 vs. 12–23 *p* = 0.001; flesh foods 6–8 vs. 12–23 *p* < 0.001 and 9–11 vs. 12–23 *p* = 0.001; vitamin A-rich fruits/vegetables 6–8 vs. 12–23 *p* < 0.001 and 9–11 vs. 12–23 *p* < 0.001; other fruits/vegetables 6–8 vs. 12–23 *p* < 0.001 and 9–11 vs. 12–23 *p* = 0.008; MDD 6–8 vs.12–23 *p* < 0.001 and 9–11 vs. 12–23 *p* < 0.001. Area: dairy products rural vs. urban *p* < 0.001; flesh foods rural vs. urban *p* < 0.001; eggs rural vs. urban *p* < 0.001; other fruits/vegetables rural vs. urban *p* < 0.001; MDD rural vs. urban *p* = 0.020. Wealth status: legumes/nuts poor vs. rich *p* = 0.023; flesh foods poor vs. rich *p* < 0.001 and medium vs. rich *p* = 0.004; eggs poor vs. rich *p* < 0.001 and medium vs. rich *p* = 0.031; other fruits/vegetables poor vs. rich *p* = 0.002; MDD poor vs. rich *p* = 0.006.

‡ Based on the child’s prior 24-h consumption of ≥5 of 8 food groups: breast milk; grains/roots/tubers; legumes/nuts; dairy products; flesh foods; eggs; vitamin A-rich fruits/vegetables; and other fruits/vegetables.

**Table 5:** Food group consumption and minimum dietary diversity by child age group, sex, area and household wealth in Senegal^†^.

	Total	6–11 mo	6–8 mo	9–11 mo	12–23 mo	Female	Male	Rural	Urban	Poor	Medium	Rich
	n (%)	n (%)	n (%)	n (%)	n (%)	n (%)	n (%)	n (%)	n (%)	n (%)	n (%)	n (%)
Breast milk	1,854 (70.0)	878 (96.0)	496 (96.1)	382 (95.7)	976 (56.4)	888 (69.7)	966 (70.4)	1,489 (70.3)	365 (69.0)	744 (74.8)	380 (69.6)	730 (65.9)
Grains, roots, tubers	2,267 (85.6)	650 (71.0)	334 (64.7)	316 (79.2)	1,617 (93.4)	1,090 (85.6)	1,177 (85.7)	1,828 (86.3)	439 (83.0)	860 (86.5)	472 (86.4)	935 (84.5)
Pulses, nuts, seeds	728 (27.5)	144 (15.7)	62 (12.0)	82 (20.6)	584 (33.7)	352 (27.6)	376 (27.4)	594 (28.0)	134 (25.3)	329 (33.1)	138 (25.3)	261 (23.6)
Dairy products	1,386 (52.4)	398 (43.5)	199 (38.6)	199 (49.9)	988 (57.0)	641 (50.3)	745 (54.3)	1,083 (51.1)	303 (57.3)	456 (45.9)	298 (54.6)	632 (57.1)
Flesh foods	1,200 (45.3)	202 (22.1)	85 (16.5)	117 (29.3)	998 (57.6)	576 (45.2)	624 (45.4)	925 (43.7)	275 (52.0)	357 (35.9)	257 (47.1)	586 (52.9)
Eggs	199 (7.5)	37 (4.0)	20 (3.9)	17 (4.3)	162 (9.4)	89 (7.0)	110 (8.0)	122 (5.8)	77 (14.6)	44 (4.4)	32 (5.9)	123 (11.1)
Vitamin A-rich fruits / veg	1,410 (53.3)	359 (39.2)	159 (30.8)	200 (50.1)	1,051 (60.7)	691 (54.2)	719 (52.4)	1,119 (52.8)	291 (55.0)	475 (47.8)	277 (50.7)	658 (59.4)
Other fruits / veg	659 (24.9)	128 (14.0)	53 (10.3)	75 (18.8)	531 (30.7)	328 (25.7)	331 (24.1)	479 (22.6)	180 (34.0)	142 (14.3)	141 (25.8)	376 (34.0)
Minimum Dietary Diversity ^‡^	816 (30.8)	171 (18.7)	64 (12.4)	107 (26.8)	645 (37.2)	384 (30.1)	432 (31.5)	619 (29.2)	197 (37.2)	236 (23.7)	174 (31.9)	406 (36.7)

† Child age: breast milk 6–8 vs. 12–23 *p* < 0.001 and 9–11 vs. 12–23 *p* < 0.001; grains/roots/tubers 6–8 vs. 9–11 *p* < 0.001, 6–8 vs. 12–23 *p* < 0.001 and 9–11 vs. 12–23 *p*< 0.001; legumes/nuts 6–8 vs. 9–11 *p* = 0.010, 6–8 vs. 12–23 *p* < 0.001 and 9–11 vs. 12–23 *p* < 0.001; dairy 6–8 vs. 9–11 *p* = 0.002, 6–8 vs. 12–23 *p* < 0.001 and 9–11 vs. 12–23 *p* = 0.025; flesh foods 6–8 vs. 9–11 *p* < 0.001, 6–8 vs. 12–23 *p* < 0.001 and 9–11 vs. 12–23 *p* < 0.001; eggs 6–8 vs. 12–23 *p* < 0.001 and 9–11 vs. 12–23 *p* = 0.001; vitamin A-rich fruits/vegetables 6–8 vs. 9–11 *p* < 0.001, 6–8 vs. 12–23 *p* < 0.001 and 9–11 vs. 12–23 *p* < 0.001; other fruits/vegetables 6–8 vs. 9–11 *p* = 0.007, 6–8 vs.12–23 *p* < 0.001 and 9–11 vs. 12–23 *p* < 0.001; MDD 6–8 vs. 9–11 *p* < 0.001, 6–8 vs. 12–23 *p* < 0.001 and 9–11 vs. 12–23 *p* < 0.001. Child sex: dairy products female vs. male *p* = 0.042. Area: dairy products rural vs. urban *p* = 0.011; flesh foods rural vs. urban *p* = 0.001; eggs rural vs. urban *p* < 0.001; other fruits/vegetables rural vs. urban *p* < 0.001; MDD rural vs. urban *p* < 0.001. Wealth status: breast milk poor vs. rich *p* < 0.001; legumes/nuts poor vs. medium *p* = 0.003 and poor vs. rich *p* < 0.001; dairy products poor vs. medium *p* = 0.003 and poor vs. rich *p* < 0.001; flesh foods poor vs. medium *p* < 0.001 and poor vs. rich *p* < 0.001; eggs poor vs. rich *p* < 0.001 and medium vs. rich *p* < 0.001; vitamin A-rich fruits/vegetables poor vs. rich *p* < 0.001 and medium vs. rich *p* = 0.002; other fruits/vegetables poor vs. medium *p* < 0.001, poor vs. rich *p* < 0.001 and medium vs. rich *p* = 0.001; MDD poor vs. medium *p* = 0.003 and poor vs. rich *p* < 0.001.

‡ Based on the child’s prior 24-h consumption of ≥5 of 8 food groups: breast milk; grains, roots and tubers; legumes and nuts; dairy products; flesh foods; eggs; vitamin A-rich fruits and vegetables; and other fruits and vegetables.

In Niger, with the exception of eggs, significant differences were observed in the consumption of all food groups and for MDD prevalence between children 6–8 and 12–23 months and between children 9–11 and 12–23 months, with higher consumption among children aged 12–23 months (Table 4). In addition, children 9–11 months had higher consumption of grains/roots/tubers/plantains compared to children 6–8 months (70.0% vs. 51.5%; *p* = 0.001). In Senegal, children 9–11 months had higher consumption of grains/roots/tubers/plantains, pulses/nuts/seeds, dairy products, flesh foods, vitamin A-rich fruits/vegetables and other fruits/vegetables and also had a higher MDD prevalence compared to those 6–8 months of age. With the exception of breast milk, children 12–23 months had higher consumption of all food groups and had a higher MDD prevalence compared to both children 6–8 and 9–11 months of age.

In Niger and Senegal, a higher percentage of children in urban, as compared to rural, areas consumed flesh foods, eggs and other fruits/vegetables (Tables 4, 5). Children in urban areas also had a higher MDD prevalence in Niger (37.5% vs. 29.5%; *p* = 0.020) and Senegal (37.2% vs. 29.2%; *p* < 0.001). In Niger, the consumption of dairy products was higher among children in rural, as compared to urban, households (52.5% vs. 31.4%; *p* < 0.001). Compared to children in the poorest households, a larger percentage of children in the wealthiest households achieved MDD in Niger (37.8% vs. 26.3%; *p* = 0.006) and Senegal (36.7% vs. 23.7%; *p* < 0.001) and also had higher intake of flesh foods, eggs and other fruits/vegetables. Conversely, in Senegal, a larger percentage of children in the poorest, as compared to richest, households were breastfed (74.8% vs. 65.9%; *p* < 0.001) and provided pulses/nuts/seeds (33.1% vs. 23.6%; *p* < 0.001) the previous day. In Côte d’Ivoire, both vitamin A-rich fruit/vegetable consumption (69.4% vs. 36.8%; *p* = 0.039) and MDD prevalence (65.3% vs. 31.6%; *p* = 0.033) were higher among children in the poorest, compared to medium wealth, households (Table 3).

In the multivariable analyses for Niger and Senegal, children 12–23 months had a higher likelihood of MDD, compared to those aged 6–11 months (Niger: aOR = 4.25; 95% CI: 2.46, 7.36; Senegal: aOR = 2.69; 95% CI: 2.15, 3.35; Table 6). In Senegal, children of mothers with a secondary or higher education were more likely to attain MDD (aOR = 1.93; 95% CI: 1.44, 2.59), as compared to children whose mothers had no formal education. Increased household wealth was also positively associated with children’s MDD in Senegal, with those in medium (aOR = 1.64; 95% CI: 1.25, 2.14) and high (aOR = 1.88; 95% CI: 1.50, 2.37) wealth households having a higher likelihood of MDD, as compared to children in the poorest households (Table 6).

**Table 6:** Multivariable associations with children’s minimum dietary diversity in Niger and Senegal.

	Niger uOR (95% CI)	Niger aOR (95% CI)	Senegal uOR (95% CI)	Senegal aOR (95% CI)
Child age 6–11 months 12–23 months	1.00 3.21 (1.95, 5.27)	1.00 4.25 (2.46, 7.36)	1.00 2.56 (2.07, 3.17)	1.00 2.69 (2.15, 3.35)
Child sex Male Female	1.00 1.51 (1.01, 2.26)	1.00 1.44 (0.93, 2.25)	1.00 0.93 (0.77, 1.12)	1.00 0.96 (0.79, 1.17)
Mother’s education None Primary Secondary / higher	1.00 1.49 (0.89, 2.51) 1.27 (0.69, 2.35)	1.00 1.61 (0.94, 2.77) 1.14 (0.58, 2.24)	1.00 1.27 (0.99, 1.62) 2.17 (1.65, 2.85)	1.00 1.19 (0.92, 1.54) 1.93 (1.44, 2.59)
Area Urban Rural	1.00 0.82 (0.55, 1.21)	1.00 1.16 (0.71, 1.90)	1.00 0.75 (0.60, 0.94)	1.00 0.89 (0.69, 1.15)
Household wealth Poor Medium Rich	1.00 1.28 (0.61, 2.71) 1.62 (1.07, 2.47)	1.00 1.01 (0.42, 2.39) 1.49 (0.90, 2.49)	1.00 1.71 (1.32, 2.21) 1.99 (1.62, 2.46)	1.00 1.64 (1.25, 2.14) 1.88 (1.50, 2.37)

uOR, unadjusted odds ratio; aOR, adjusted odds ratio. Reference categories: 6–11 months, male, non-educated mothers, urban, poor. (Reference population is mothers).

## 4 Discussion

In this study, we examined the variety of foods consumed by infants and young children in three West African settings. Low MDD prevalence was observed in all three countries, with one-third of children having an adequately diversified diet in Niger and Senegal. Starchy staple foods were most commonly consumed (≥ 75%) in all countries and consumption of animal-source foods varied, with especially low intake of flesh foods in Niger and low egg consumption across all three countries. This is consistent with evidence indicating a reliance on grains, roots or tubers and lower intake of high-quality protein sources where access to such cost-prohibitive foods is limited (7–12). In all three countries, about half of the children consumed vitamin A-rich fruits and/or vegetables, which could potentially be due to nutrition-focused community education emphasizing the importance of vitamin A-containing foods for young children and access to these foods in the study areas. This was especially likely in Côte d’Ivoire where the surveyed areas were targeted for an agro-nutrition project that included promoting the cultivation of orange-fleshed sweet potatoes.

Breastfeeding was more prevalent for children aged 6–11 months, compared to 12–23 months, in all countries. This was expected as the decline in continued breastfeeding after 6–12 months is common across diverse settings (17). The ~10% higher prevalence of breastfeeding in the poorest, as compared to wealthiest, households in Senegal may be due to these mothers having less exposure to media influences promoting undesirable foods for infants and young children and/or less income to purchase such products. Also, available evidence for continued breastfeeding practices in Western Africa indicates negative associations between wealth and breastfeeding duration (18).

In all three countries, children aged 12–23 months had higher consumption of starchy staples, pulses/nuts/seeds, flesh foods, vitamin A-rich fruits/vegetables and other fruits/vegetables than infants 6–11 months of age. Accordingly, MDD prevalence was 30% higher for children ≥12 months in Côte d’Ivoire and was more than twice as high for these children, compared to younger infants, in Niger and Senegal. The higher consumption of starchy staples in Niger and Senegal and higher MDD prevalence in Senegal among older infants 9–11 months, compared to 6–8 months, are also noteworthy. Older children eating more diverse diets is a common pattern and likely reflects children’s growth and increased gastric capacity, as well as other factors such as mothers’ hesitancy to feed younger children certain foods and traditional beliefs surrounding appropriate foods and feeding practices for very young children, as has been observed for breastfeeding and complementary feeding in SSA (19, 20). In a recent study assessing MDD among children aged 6–23 months in The Gambia, Liberia and Rwanda, children 12–17 months (OR = 1.96; 95% CI: 1.61, 2.39) and 18–23 months (OR = 1.92; 95% CI: 1.58, 2.33) were almost twice as likely to attain MDD, as compared to those 6–11 months of age (21). Furthermore, a study involving 2010–2020 DHS data for 33 SSA countries revealed that children aged 12–23 months were less likely to have inadequate MDD, as compared to infants 6–8 months of age (OR = 0.31; 95% CI: 0.29, 0.33) (12).

Findings from the recent Côte d’Ivoire DHS revealed age-specific increases in the consumption of eggs and/or other animal-source foods from 32% at 6–11 months to 77% at 18–23 months (22). In addition, the survey reported 70% of children aged 6–11 months did not consume any fruits or vegetables the previous day, compared to 30% at 18–23 months. In our study, the higher consumption of animal-source foods and fruits/vegetables and overall greater dietary diversity, particularly for children in the poorest households, in Côte d’Ivoire are notable. As mentioned previously, these findings are presumably due to the study population being part of a broader agriculture and nutrition-focused project. Nevertheless, identifying other factors that may have contributed to children’s > 50% consumption of six food groups in these solely rural areas merits further investigation.

In Niger and Senegal, higher flesh food consumption and MDD prevalence were observed among children in urban areas and in the wealthiest households. These were not unexpected findings as socio-economically advantaged populations are more likely to have better physical and monetary access to a greater variety of high-quality nutritious foods. Pooled data from the above-referenced study (12) involving 33 SSA countries highlighted the increased likelihood of inadequate MDD among children in rural, as compared to urban, areas (OR = 1.29; 95% CI: 1.23, 1.36) and showed that increased household wealth was protective for children’s dietary diversity. In our study, maternal education was positively associated with children’s MDD in Senegal and has been shown to be a key predictor of IYCF practices in most low-resource settings, including in West Africa (23–27).

Our MDD estimates are considerably higher than national estimates reported for Côte d’Ivoire and Niger (22, 28). This is likely due to differing survey methods and seasonal variations in food availability. However, our study findings are consistent with other evidence showing infants from rural areas, uneducated families and low-income households typically have poorer dietary diversity (11, 12, 21, 23–28). Therefore, to improve children’s nutritional status, multisectoral collaboration is essential to promote awareness and access to nutritious foods and good complementary feeding during the 6-23-month period.

Lastly, the low consumption of sugary drinks in all three countries and for packaged chips in Côte d’Ivoire and Niger are encouraging, given the increasing availability of unhealthy snacks and beverages targeted to young children in low-income countries (29). Our findings contrast with the higher consumption of sugary drinks and “junk” foods reported in the 2021 DHS for Côte d’Ivoire (22). Though reasons for the low intake of these items in our study areas are not known, cost considerations and availability may be relevant in these settings. The much higher consumption of packaged chips in Senegal was unexpected and merits further exploration, particularly in light of the fact that 80% of the sample population resided in rural areas.

Interpretations and inferences based on our findings should take into account some key aspects of our study. The survey timeframe in each country should be considered, given the seasonal fluctuations in accessing certain foods during lean and post-harvest periods. As a limitation of 24-h recall methodology is the lack of data on food quantities consumed, it was not possible to know whether the amounts children consumed of each food category were sufficient for age-specific nutritional adequacy. We explored the influence of key predictors of children’s dietary intake. Other factors that were not assessed, such as household food security and caregiver knowledge of IYCF practices, should be considered as likely contributors to child feeding practices. Lastly, the cross-sectional survey design precludes causal inferences and, as for all self-reported data, recall and social desirability biases may have occurred. Despite these limitations, our results are based on representative data from large samples in Niger and Senegal and standard DQQ methods were utilized in all countries. The use of the novel DQQ methodology is a key strength of our study as it allows for an understanding of both health-promoting and unhealthy food contributors to daily diets and, thereby, adds valuable data for country nutrition program planning. Helen Keller Intl is an early adopter of this innovative methodology which is now also being incorporated into the DHS model. Moreover, these were primary data collected by Helen Keller Intl teams in each country with robust monitoring systems and provisions for data quality assurance.

In this study, we examined the variety of foods consumed by infants and young children and associated factors in three countries in Western Africa. Our findings have significant public health implications for these settings as they indicate most children are not receiving an adequately diversified diet and, consequently, are unlikely meeting their daily essential nutrient requirements. Given the foundational importance of nutrition during this period of life, our study highlights the urgent continuing need for increased investments in strategies that support optimal complementary feeding practices. This should include motivational counseling for caretakers about the value of a varied diet in achieving aspirations for their children, as well as increasing access to and affordability of diverse and nutritious foods as part of well-designed social and behavioral change strategies. Tools such as the Diet Quality Questionnaire should be considered for monitoring and evaluating progress toward improving children’s dietary quality with more diversified foods as a cornerstone of intersectoral efforts to reduce child malnutrition in Western Africa.

## Data availability statement

The raw data supporting the conclusions of this article will be made available by the authors, without undue reservation.

## Ethics statement

The studies involving humans were approved by Comité National d’Ethique des Sciences de la Vie et de la Santé in Côte d’Ivoire, Comité National d’Ethique pour la Recherche en Santé in Niger, and Comité National d’Ethique pour la Recherche en Santé in Senegal. The studies were conducted in accordance with the local legislation and institutional requirements. Written informed consent was obtained in Niger and Senegal and verbal informed consent was obtained from participants in Côte d’Ivoire.

## Author contributions

AJ: Data curation, Formal analysis, Methodology, Writing – original draft, Writing – review & editing. MMB: Supervision, Writing – original draft, Writing – review & editing. DD: Writing – review & editing. FN: Supervision, Writing – review & editing. AK: Supervision, Writing – review & editing. AL: Supervision, Writing – review & editing. KK: Supervision, Writing – review & editing. MB: Supervision, Writing – review & editing. MY: Supervision, Writing – review & editing. RD: Conceptualization, Data curation, Methodology, Supervision, Writing – original draft, Writing – review & editing, Formal analysis.
